# Longitudinal Changes in Creatinine Filtration as a Novel Index of Muscle Mass and Mortality Risk

**DOI:** 10.1093/geroni/igaf122.1217

**Published:** 2025-12-31

**Authors:** Shoshana Ballew, Aditya Surapaneni, Jordan Weiss, Hubert Leo, Morgan Grams, Marcus Goncalves, Josef Coresh

**Affiliations:** New York University, New York, New York, United States; New York University, New York, New York, United States; NYU Grossman School of Medicine, New York, New York, United States; New York University, New York, New York, United States; New York University, New York, New York, United States; New York University, New York, New York, United States; New York University, New York, New York, United States

## Abstract

Sarcopenia, defined by low muscle mass, is common among older adults and is associated with adverse outcomes. Quantifying muscle mass is challenging in routine clinical practice as the gold standard measures are expensive, cumbersome, and time consuming. A single timepoint of estimated glomerular filtration of creatinine (eGFcr=eGFR using serum cystatin C x serum creatinine) as an index of muscle mass has been shown to be associated with frailty, mortality, and muscle imaging measurement. Cox regression was used to estimate associations of change in eGFcr from visit 5 (2011–2013) to visit 6 (2016-2017) of a community-based prospective cohort, the Atherosclerosis Risk in Communities (ARIC) Study, with mortality. Models were adjusted for demographics, clinical variables, and comorbid conditions. Among the 2943 participants (average age 75 years, 56% women), 2210 participants had a decline in eGFcr (76.8% of men and 73.4% of women) and 280 participants had a decrease of > 5% per year (9.7% in men and 9.3% in women). There were 407 deaths over 4.5 years of follow-up. In models adjusted for baseline covariates, women with a decrease in eGFcr had an increased risk of mortality (HR 1.01 CI: 0.96-1.06 and HR 1.08 CI: 1.03-1.37 per 1% decline in eGFcr in men and women, respectively). When accounting for the last eGFcr measure, the change was no longer significant. The eGFcr can be used in research and clinical practice to assess muscle mass in older adults, and the trajectories over time may be informative for risk when considering prevention of muscle loss.

